# (μ-5-Carb­oxy-1*H*-imidazole-4-carboxyl­ato-κ^4^ *N* ^1^,*O* ^5^:*N* ^3^,*O* ^4^)bis­[amminesilver(I)]

**DOI:** 10.1107/S1600536809045747

**Published:** 2009-11-07

**Authors:** Rui-Sha Zhou, Jiang-Feng Song

**Affiliations:** a Department of Chemistry, North University of China, Taiyuan Shanxi 030051, People’s Republic of China

## Abstract

In the title compound, [Ag_2_(C_5_H_2_N_2_O_4_)(NH_3_)_2_], each of the two Ag^I^ atoms is coordinated by two N atoms from an ammonia mol­ecule and a 5-carb­oxy-1*H*-imidazole-4-carboxyl­ate ligand in an almost linear geometry, and by one carboxyl­ate O atom with a weak inter­action. The Ag atoms are assembled into a linear tetra­mer through Ag⋯Ag inter­actions. Each Ag tetra­mer is linked by four 5-carb­oxy-1*H*-imidazole-4-carboxyl­ate ligands, forming a puckered chain. The complex involves a strong intra­molecular O—H⋯O hydrogen bond.

## Related literature

For general background to coordination polymers, see: Ferey (2008 ▶); Ma *et al.* (2009 ▶); Moulton & Zaworotko (2001 ▶); Tranchemontagne *et al.* (2009 ▶). For related structures with the 4,5-imidazole­dicarboxylic acid ligand, see: Caudle *et al.* (1997 ▶); Fang & Zhang (2006 ▶); Han *et al.* (2005 ▶); Zhong *et al.* (2006 ▶).
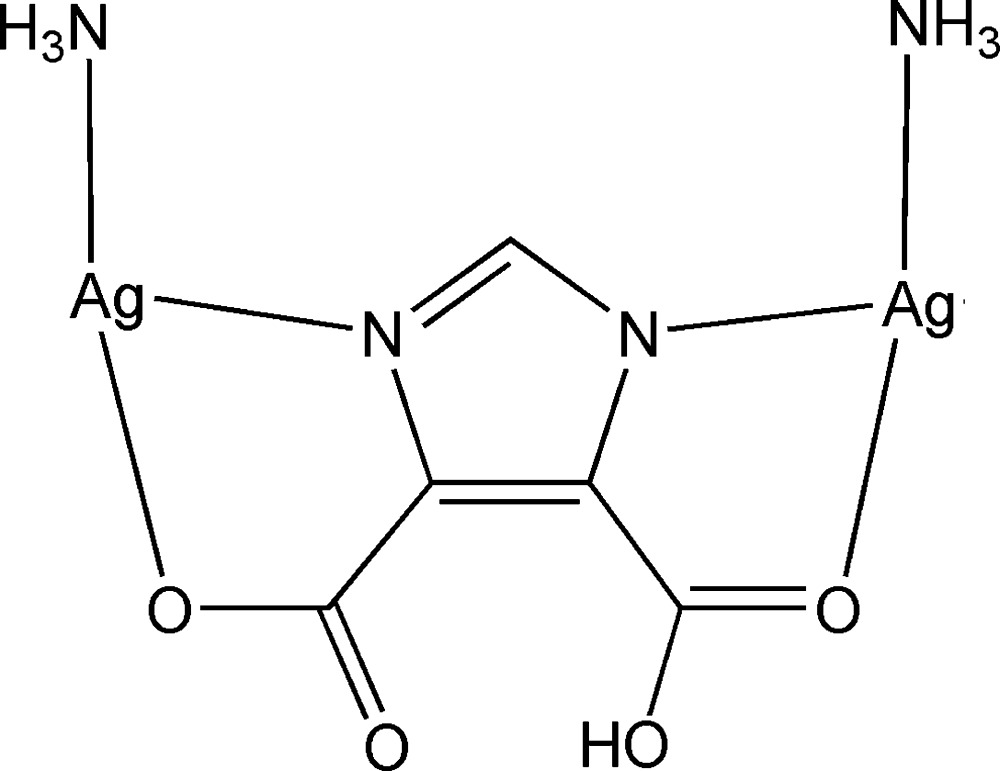


## Experimental

### 

#### Crystal data

[Ag_2_(C_5_H_2_N_2_O_4_)(NH_3_)_2_]
*M* *_r_* = 403.89
Monoclinic, 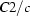

*a* = 18.3800 (12) Å
*b* = 8.3243 (5) Å
*c* = 13.6696 (8) Å
β = 113.160 (1)°
*V* = 1922.9 (2) Å^3^
*Z* = 8
Mo *K*α radiation
μ = 4.07 mm^−1^
*T* = 190 K
0.35 × 0.25 × 0.10 mm

#### Data collection

Bruker SMART APEX CCD diffractometer
Absorption correction: multi-scan (*SADABS*; Bruker, 2001 ▶) *T*_min_ = 0.330, *T*_max_ = 0.686
5211 measured reflections
1911 independent reflections
1721 reflections with *I* > 2σ(*I*)
*R*_int_ = 0.019

#### Refinement

*R*[*F* ^2^ > 2σ(*F* ^2^)] = 0.018
*wR*(*F* ^2^) = 0.044
*S* = 1.05
1911 reflections
168 parameters
H atoms treated by a mixture of independent and constrained refinement
Δρ_max_ = 0.45 e Å^−3^
Δρ_min_ = −0.50 e Å^−3^

### 

Data collection: *SMART* (Bruker, 2007 ▶); cell refinement: *SAINT* (Bruker, 2007 ▶); data reduction: *SAINT*; program(s) used to solve structure: *SHELXS97* (Sheldrick, 2008 ▶); program(s) used to refine structure: *SHELXL97* (Sheldrick, 2008 ▶); molecular graphics: *DIAMOND* (Brandenburg, 1999 ▶); software used to prepare material for publication: *SHELXTL* (Sheldrick, 2008 ▶).

## Supplementary Material

Crystal structure: contains datablocks I, global. DOI: 10.1107/S1600536809045747/hy2243sup1.cif

Structure factors: contains datablocks I. DOI: 10.1107/S1600536809045747/hy2243Isup2.hkl

Additional supplementary materials:  crystallographic information; 3D view; checkCIF report

## Figures and Tables

**Table 1:** Selected bond lengths (Å)

Ag1—N1	2.134 (2)
Ag1—N4	2.123 (3)
Ag1—O4	2.628 (2)
Ag2—N2	2.132 (2)
Ag2—N3	2.133 (3)
Ag2—O1	2.607 (2)
Ag1⋯Ag1^i^	2.9916 (5)
Ag1⋯Ag2^ii^	3.0021 (4)

Symmetry codes: (i) 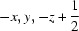; (ii) 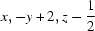.

**Table 2:** Hydrogen-bond geometry (Å, °)

*D*—H⋯*A*	*D*—H	H⋯*A*	*D*⋯*A*	*D*—H⋯*A*
O2—H2⋯O3	1.19 (8)	1.26 (8)	2.448 (3)	172 (6)

**Fig. 1.**
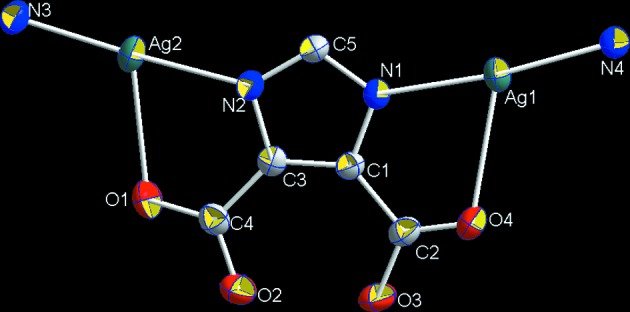
The molecular structure of the title compound. Displacement ellipsoids are drawn at the 50% probability level.

**Fig. 2.**
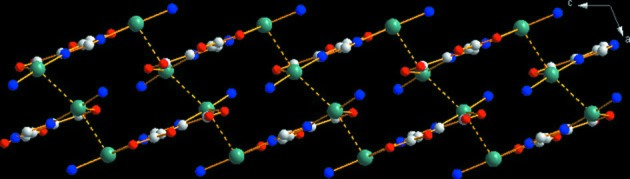
The one-dimensional puckered chain assembled by Ag tetramers and 4,5-imidazoledicarboxylate ligands. Dashed lines denote Ag···Ag interactions.

## supplementary crystallographic information

### Comment

Coordination polymers are rapidly increasing because of their intriguing structures and wide potential applications as functional materials (Ferey, 2008; Ma *et al.*, 2009; Moulton & Zaworotko, 2001; Tranchemontagne *et al.*, 2009). Complexes with 4,5-imidazoledicarboxylic acid ligand have been recently reported (Caudle *et al.*, 1997; Fang & Zhang, 2006; Zhong *et al.*, 2006). The title compound is a new Ag^I^ complex built with 4,5-imidazoledicarboxylic acid ligand.

The asymmetric unit consists of two crystallographically independent Ag^I^ atoms, one partly deprotonated 4,5-imidazoledicarboxylate ligand and two ammonia molecules (Fig. 1). Each Ag^I^ atom is bonded to an ammonia molecule and one N atom and one O atom from the 4,5-imidazoledicarboxylate ligand in a chelating coordination mode. The Ag—N distances range from 2.123 (3) to 2.134 (2) Å (Table 1), and the N—Ag—N bond angles range from 173.75 (11) to 177.4 (11)°. It is worth to note that the bond distances of Ag—O [2.628 (2) and 2.607 (2) Å] are much longer than that observed in the reported silver carboxylate complexes (Han *et al.*, 2005), indicating weak interaction between the Ag^I^ atom and the carboxylate O atom. Ag1 and Ag2 are assembled into an Ag2···Ag1···Ag1···Ag2 linear tetramer through the Ag···Ag interactions (Fig. 2) [Ag1···Ag1^i^ = 2.9916 (5) and Ag1···Ag2^ii^ = 3.0021 (4) Å; symmetry codes: (i) -*x*, *y*, 1/2 - *z*; (ii) *x*, 2 - *y*, -1/2 + *z*]. Moreover, there exists a strong hydrogen bond (O2—H2···O3) in the complex molecule (Table 2).

### Experimental

A solution of 4,5-imidazoledicarboxylic acid (6.5 mg, 0.05 mmol) in 2 ml water containing triethylamine (14 µl, 1 mmol) was directly mixed with a solution of AgNO_3_ in 2 ml of 25% aqueous ammonia. The resulting yellow solution was allowed to slowly crystalize at room temperature in dark. After one week, brown single crystals were collected by filtration, washed with small amounts of water and dried in air.

### Refinement

H atoms were located in a difference Fourier map and refined isotropically.

### Crystal data

**Table:**

[Ag_2_(C_5_H_2_N_2_O_4_)(NH_3_)_2_]	*F*(000) = 1536
*M**_r_* = 403.89	*D*_x_ = 2.790 Mg m^−^^3^
Monoclinic, *C*2/*c*	Mo *K*α radiation, λ = 0.71073 Å
Hall symbol: -C 2yc	Cell parameters from 1780 reflections
*a* = 18.3800 (12) Å	θ = 2.4–26.1°
*b* = 8.3243 (5) Å	µ = 4.07 mm^−^^1^
*c* = 13.6696 (8) Å	*T* = 190 K
β = 113.160 (1)°	Block, brown
*V* = 1922.9 (2) Å^3^	0.35 × 0.25 × 0.10 mm
*Z* = 8	

### Data collection

**Table:**

Bruker SMART APEX CCD diffractometer	1911 independent reflections
Radiation source: fine-focus sealed tube	1721 reflections with *I* > 2σ(*I*)
graphite	*R*_int_ = 0.019
φ and ω scans	θ_max_ = 26.1°, θ_min_ = 2.4°
Absorption correction: multi-scan (*SADABS*; Bruker, 2001)	*h* = −22→18
*T*_min_ = 0.330, *T*_max_ = 0.686	*k* = −10→9
5211 measured reflections	*l* = −16→16

### Refinement

**Table:**

Refinement on *F*^2^	Primary atom site location: structure-invariant direct methods
Least-squares matrix: full	Secondary atom site location: difference Fourier map
*R*[*F*^2^ > 2σ(*F*^2^)] = 0.018	Hydrogen site location: inferred from neighbouring sites
*wR*(*F*^2^) = 0.044	H atoms treated by a mixture of independent and constrained refinement
*S* = 1.05	*w* = 1/[σ^2^(*F*_o_^2^) + (0.02*P*)^2^ + 2.4121*P*] where *P* = (*F*_o_^2^ + 2*F*_c_^2^)/3
1911 reflections	(Δ/σ)_max_ = 0.001
168 parameters	Δρ_max_ = 0.45 e Å^−^^3^
0 restraints	Δρ_min_ = −0.50 e Å^−^^3^

### Fractional atomic coordinates and isotropic or equivalent isotropic displacement parameters (Å^2^)

**Table:**

	*x*	*y*	*z*	*U*_iso_*/*U*_eq_	
Ag2	0.206133 (14)	0.79951 (3)	0.660249 (17)	0.03620 (8)	
O1	0.17397 (15)	0.5366 (3)	0.54751 (18)	0.0476 (6)	
N2	0.15598 (14)	0.8616 (3)	0.49573 (18)	0.0306 (5)	
Ag1	0.060181 (14)	1.13414 (3)	0.201582 (18)	0.03346 (8)	
C1	0.11079 (17)	0.8155 (3)	0.3218 (2)	0.0269 (6)	
C2	0.08372 (18)	0.7496 (4)	0.2126 (2)	0.0322 (7)	
C3	0.13893 (16)	0.7428 (4)	0.4199 (2)	0.0275 (6)	
C4	0.15067 (17)	0.5712 (4)	0.4527 (2)	0.0323 (7)	
C5	0.13741 (19)	0.9977 (4)	0.4412 (2)	0.0332 (7)	
N1	0.11022 (14)	0.9785 (3)	0.33560 (18)	0.0302 (5)	
N3	0.25466 (19)	0.7478 (4)	0.8265 (2)	0.0366 (6)	
N4	0.0094 (2)	1.3080 (4)	0.0800 (2)	0.0368 (6)	
O2	0.13419 (14)	0.4656 (3)	0.37827 (19)	0.0436 (6)	
O3	0.08142 (16)	0.5948 (3)	0.20328 (18)	0.0480 (6)	
O4	0.06587 (15)	0.8395 (3)	0.13591 (16)	0.0441 (6)	
H1	0.1399 (19)	1.094 (4)	0.467 (3)	0.040 (9)*	
H2	0.112 (4)	0.523 (9)	0.291 (6)	0.17 (3)*	
H3	0.275 (2)	0.836 (5)	0.869 (3)	0.062 (12)*	
H4	0.300 (2)	0.686 (5)	0.843 (3)	0.058 (12)*	
H5	0.218 (2)	0.696 (4)	0.844 (3)	0.042 (10)*	
H6	0.041 (2)	1.385 (5)	0.078 (3)	0.060 (13)*	
H7	−0.034 (3)	1.348 (5)	0.083 (3)	0.060 (13)*	
H8	−0.004 (3)	1.271 (6)	0.024 (4)	0.073 (16)*	

### Atomic displacement parameters (Å^2^)

**Table:**

	*U*^11^	*U*^22^	*U*^33^	*U*^12^	*U*^13^	*U*^23^
Ag2	0.03743 (15)	0.04560 (16)	0.02245 (12)	0.00084 (10)	0.00842 (10)	0.00281 (10)
O1	0.0657 (16)	0.0373 (12)	0.0384 (13)	0.0061 (11)	0.0189 (12)	0.0123 (10)
N2	0.0315 (13)	0.0331 (13)	0.0236 (12)	0.0025 (10)	0.0072 (11)	−0.0013 (10)
Ag1	0.03572 (14)	0.03391 (14)	0.02914 (13)	0.00315 (10)	0.01104 (10)	0.00742 (9)
C1	0.0292 (15)	0.0262 (14)	0.0257 (14)	−0.0018 (11)	0.0112 (12)	−0.0028 (11)
C2	0.0312 (16)	0.0350 (16)	0.0281 (15)	−0.0025 (13)	0.0091 (13)	−0.0058 (13)
C3	0.0238 (14)	0.0324 (15)	0.0271 (14)	0.0005 (11)	0.0110 (12)	0.0006 (12)
C4	0.0318 (16)	0.0319 (16)	0.0357 (16)	0.0040 (12)	0.0159 (13)	0.0032 (13)
C5	0.0407 (18)	0.0283 (16)	0.0256 (14)	0.0030 (13)	0.0076 (13)	−0.0036 (12)
N1	0.0348 (14)	0.0288 (12)	0.0236 (11)	0.0020 (10)	0.0078 (11)	0.0023 (10)
N3	0.0409 (17)	0.0394 (16)	0.0255 (13)	−0.0030 (13)	0.0087 (13)	−0.0003 (12)
N4	0.0449 (18)	0.0308 (15)	0.0293 (15)	0.0008 (13)	0.0089 (14)	0.0015 (12)
O2	0.0585 (15)	0.0284 (11)	0.0447 (13)	0.0004 (10)	0.0212 (12)	−0.0026 (10)
O3	0.0710 (17)	0.0340 (12)	0.0370 (12)	−0.0047 (12)	0.0189 (12)	−0.0112 (10)
O4	0.0606 (15)	0.0428 (13)	0.0230 (11)	0.0044 (11)	0.0100 (11)	−0.0014 (10)

### Geometric parameters (Å, °)

**Table:**

Ag1—N1	2.134 (2)	C2—O4	1.223 (4)
Ag1—N4	2.123 (3)	C2—O3	1.294 (4)
Ag1—O4	2.628 (2)	C3—C4	1.487 (4)
Ag2—N2	2.132 (2)	C4—O2	1.287 (4)
Ag2—N3	2.133 (3)	C5—N1	1.338 (4)
Ag2—O1	2.607 (2)	C5—H1	0.87 (4)
Ag1—Ag1^i^	2.9916 (5)	N3—H3	0.92 (4)
Ag1—Ag2^ii^	3.0021 (4)	N3—H4	0.93 (4)
O1—C4	1.228 (4)	N3—H5	0.90 (4)
N2—C5	1.325 (4)	N4—H6	0.87 (4)
N2—C3	1.377 (4)	N4—H7	0.87 (4)
C1—N1	1.371 (4)	N4—H8	0.77 (5)
C1—C3	1.373 (4)	O2—H2	1.19 (8)
C1—C2	1.481 (4)	O3—H2	1.26 (8)
			
N2—Ag2—N3	177.40 (11)	C1—C3—C4	132.2 (3)
N2—Ag2—O1	71.12 (8)	N2—C3—C4	120.0 (2)
N3—Ag2—O1	111.27 (10)	O1—C4—O2	123.4 (3)
N2—Ag2—Ag1^iii^	96.10 (7)	O1—C4—C3	119.5 (3)
N3—Ag2—Ag1^iii^	82.35 (9)	O2—C4—C3	117.1 (3)
O1—Ag2—Ag1^iii^	104.71 (6)	N2—C5—N1	114.1 (3)
C4—O1—Ag2	109.3 (2)	N2—C5—H1	127 (2)
C5—N2—C3	105.0 (2)	N1—C5—H1	119 (2)
C5—N2—Ag2	135.2 (2)	C5—N1—C1	104.3 (2)
C3—N2—Ag2	119.74 (19)	C5—N1—Ag1	134.8 (2)
N4—Ag1—N1	173.75 (11)	C1—N1—Ag1	120.49 (18)
N4—Ag1—O4	115.67 (10)	Ag2—N3—H3	114 (3)
N1—Ag1—O4	70.43 (8)	Ag2—N3—H4	109 (2)
N4—Ag1—Ag1^i^	99.91 (10)	H3—N3—H4	101 (3)
N1—Ag1—Ag1^i^	76.36 (7)	Ag2—N3—H5	109 (2)
O4—Ag1—Ag1^i^	106.67 (5)	H3—N3—H5	112 (3)
N4—Ag1—Ag2^ii^	82.99 (10)	H4—N3—H5	111 (3)
N1—Ag1—Ag2^ii^	99.09 (7)	Ag1—N4—H6	115 (3)
O4—Ag1—Ag2^ii^	87.07 (6)	Ag1—N4—H7	111 (3)
Ag1^i^—Ag1—Ag2^ii^	162.610 (12)	H6—N4—H7	111 (4)
N1—C1—C3	108.8 (2)	Ag1—N4—H8	112 (4)
N1—C1—C2	119.1 (2)	H6—N4—H8	103 (4)
C3—C1—C2	132.0 (3)	H7—N4—H8	104 (4)
O4—C2—O3	122.5 (3)	C4—O2—H2	113 (3)
O4—C2—C1	120.6 (3)	C2—O3—H2	113 (3)
O3—C2—C1	116.9 (3)	C2—O4—Ag1	108.15 (19)
C1—C3—N2	107.8 (2)		

Symmetry codes: (i) −*x*, *y*, −*z*+1/2; (ii) *x*, −*y*+2, *z*−1/2; (iii) *x*, −*y*+2, *z*+1/2.

### Hydrogen-bond geometry (Å, °)

**Table:**

*D*—H···*A*	*D*—H	H···*A*	*D*···*A*	*D*—H···*A*
O2—H2···O3	1.19 (8)	1.26 (8)	2.448 (3)	172 (6)
